# An update on the genetics of age-related macular degeneration

**Published:** 2007-02-07

**Authors:** Hendrik P.N. Scholl, Monika Fleckenstein, Peter Charbel Issa, Claudia Keilhauer, Frank G. Holz, Bernhard H.F. Weber

**Affiliations:** 1 Department of Ophthalmology, University of Bonn, Bonn, Germany; 2 Department of Ophthalmology, University of Würzburg, Germany; 3 Institute for Human Genetics, University of Regensburg, Germany

## Abstract

Age-related macular degeneration (AMD) is a genetically complex disorder of the photoreceptor-RPE-Bruch's membrane-choriocapillaris complex. Family and twin studies have shown that the susceptibility for this disease is genetically influenced. The heritability has been estimated to be up to 71%. Linkage and association studies have identified several chromosomal regions that are likely to contain susceptibility loci with strongest evidence found on chromosome 1q31 and 10q26. Variants in the complement factor H (CFH) gene have been shown by several independent studies to be associated with an increased risk for AMD in Caucasian populations. These findings imply that the innate immune system may play a significant role in AMD pathogenesis. The *LOC387715*/*HTRA1* locus within 10q26 has been identified as a second major locus contributing to AMD pathogenesis. The two late forms of AMD, choroidal neovascularization and geographic atrophy, have not been found to be different in risk allele distribution. Variants within *CFH* and *LOC387715*/*HTRA1* may contribute to the increased risk of late AMD largely through their impact on precursors, such as drusen and/or other RPE/Bruch's membrane changes. Considering variants at *CFH*, *LOC387715*/*HTRA1* and complement component 2-complement factor B (*C2-FB*), high-risk homozygotes at all three loci may have a 250-fold increased risk compared to baseline. However, the identification of genetic factors has not resulted in therapeutic strategies to modify the disease so far and additional genetic and environmental factors are yet to be discovered in order to influence the onset and the progression of AMD.

## Introduction

### Genetic influence on age-related macular degeneration

AMD is a genetically complex disorder of the photoreceptor-retinal pigment epithelium (RPE)-Bruch's membrane-choriocapillaris complex [1-4]. Early age-related macular degeneration (AMD) is characterized by areas of increased pigment or hyperpigmentation (in the outer retina or choroid) and/or areas of depigmentation or hypopigmentation of the RPE, associated with intermediate or soft drusen [5]. Late AMD includes geographic atrophy (GA) and choroidal neovascularization (CNV). The latter includes any of the following features: subretinal neovascular membranes, intraretinal or subretinal scars, RPE and neurosensory retinal detachments, hard exudates, and retinal hemorrhages [5].

Late AMD is now the most common cause of untreatable blindness in the Western world, with a prevalence of 0.05% before the age of 50 years and 11.8% after 80 years of age [6]. Unless effective methods for prevention and treatment are found, the prevalence of AMD is expected to double in the coming decades due to an expected demographic shift towards aging populations [6].

The genetic influence on AMD is well known from family and twin studies [7-14]. First-degree relatives of patients with AMD, as compared with first-degree relatives in families without the disorder, are at increased risk (odds ratio, 2.4) for the condition [10], are affected at a younger age [13,15], and have an increased lifetime risk of late AMD (risk ratio, 4.2) [13].

In order to determine the relative contributions of heredity and environment to the etiology of AMD, Seddon and co-workers performed a population-based twin study of AMD including both concordant/discordant and monozygotic/dizygotic sibling pairs [16]. Heritability estimates for AMD were significant and ranged from 46% to 71%. These results underscore the need to pursue the search for AMD-related genes, despite the initial difficulties encountered with genetic analyses of a complex disease with late onset.

### Analysis of candidate genes for age-related macular degeneration

The progress made within the last decade by studying hereditary retinal dystrophies has offered some investigative leads to further study AMD genetics. The similarities that exist between the phenotypic expression in the hereditary early onset diseases and some of the later onset complex traits as seen in AMD suggests a potential involvement of such candidate genes in AMD pathogenesis. In addition, candidate genes were identified based on linkage study results (positional criteria) and knowledge about gene function (functional criteria). However, this approach has not led to a breakthrough (with the exception of complement factor B (FB) and complement component 2 (C2), see below). Table 1 summarizes candidate genes with negative (i.e. no involvement in AMD pathogenesis) results to date [17].

**Table 1:** Candidate gene studies for age-related macular degeneration.

**Chromosome**	**Gene**
1	ADPRT1, EPHX1, GLRX2, LAMC1, LamC2, LAMB3, OCLM, PRELP, RGS16, TGFB2
2	EFEMP1 (Fibulin 3), GPR75, IL1A, Fibulin 2, GPX1
3	IMPG2
6	RDS
7	AhR
8	NAT2
10	CYP2E1
11	CAT, Fibulin 4, VMD2
12,	A2M, MGST1
14	CKB
15	CYP1A1, CYP1A2
17	APOH, ITGB4
22	CYP2D6, Fibulin 1, TIMP3

Genes with negative results to date. For references, see Haddad et al. [17].

For other genes, some evidence of an association with AMD has been shown. Genes with at least one result of positive association are summarized in Table 2 [17]. However, variations in those genes either account for only a small fraction of AMD susceptibility or the results are inconclusive.

**Table 2:** Candidate gene studies for age-related macular degeneration.

**Chromosome**	**Gene**
1	ABCA4, HEMICENTIN (Fibulin 6)
3	CX3CR1
6	HLA genes, VEGF, ELOVL4, SOD2
7	PON1
9	VLDLR, TLR4
12	LRP6
14	Fibulin 5
17	ACE
19	APOE
20	CST3, MMP9

Genes with at least one positive result to date. For a comprehensive review of these genes including references, see Haddad et al. [17].

*Fibulin5* represents an example of genes that probably account for only a small fraction of genetic susceptibility to AMD. Stone and colleagues found that the disruption of a gene of the same gene family, *EFEMP1* (*Fibulin3*), is linked to Malattia leventinese/Doyne honeycomb retinal dystrophy [18]. This disorder is characterized by confluent drusen accumulation beneath the RPE, an early hallmark of AMD. EFEMP1 is an extracellular matrix protein. The interaction with other extracellular matrix proteins, such as adhesion molecules, collagens, elastins, fibronectins, laminins, tenascins, hemicentins and vitronectins, suggests an entire group of genes as possible candidates for involvement in drusen formation [18]. Later, the same group systematically evaluated five fibulin genes in a large series of patients with AMD. They demonstrated a significant association between sequence variations in *fibulin5* and AMD. However, missense mutations in *fibulin5* were estimated to account for only 1.7% of patients with AMD [19].

**Figure 1:**
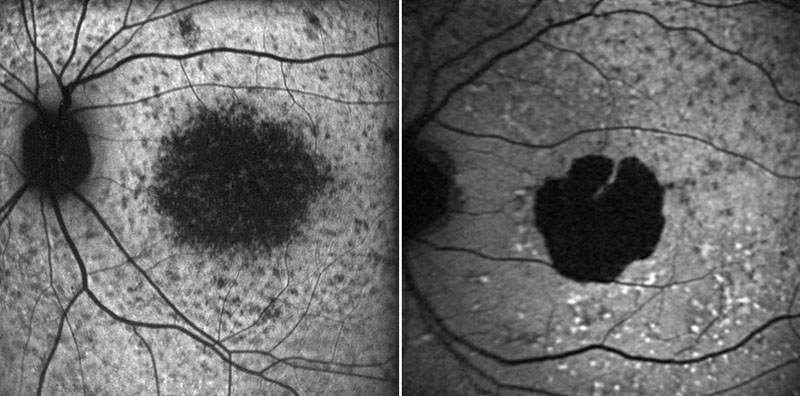
Phenotyping by means of fundus autofluorescence imaging. Fundus autofluorescence images obtained with a cSLO (Heidelberg retina angiograph, HRA 2, Heidelberg Engineering, Dossenheim, Germany) according to a standard operating procedure. Left: Patient diagnosed with Stargardt's macular dystrophy (age, 17 years); right: patient diagnosed with atrophic AMD (GA) and a fundus autofluorescence pattern "diffuse-fine granular with peripheral punctate spots" according to Bindewald et al. [34] (age: 71 years).

The photoreceptor cell-specific ATP-binding cassette transporter (*ABCA4*) gene was identified in 1997 and found to be mutated in patients with Stargardt's macular dystrophy [20]. *ABCA4* has been evaluated as a possible cause for other diseases with similar pathology in the macula including AMD. Two studies by Allikmets and co-workers provided evidence for an association between *ABCA4* polymorphisms and AMD [21,22]. While other studies provided support [23,24], a number of studies failed to confirm an association of *ABCA4* with AMD [25-32]. The number of patients and controls included in the latter studies appears large enough to rule out a major contribution of mutant *ABCA4* alleles in the predisposition to AMD; however, they may not be sufficient to allow minor effects to be discerned. This makes it extremely difficult to determine the significance of individual mutant *ABCA4* alleles in the predisposition to AMD, particularly those which are present in low frequency in the general population. The phenotypic similarities between typical juvenile Stargardt's macular dystrophy and some forms of late atrophic AMD suggest that refined phenotyping may be of value in discerning between the two conditions. Figure 1 shows fundus autofluorescence images of a patient with Stargardt's macular dystrophy (age, 17 years) and a patient with GA due to AMD (age, 71 years).

The impact of fundus autofluorescence imaging on more precise phenotyping and its potential as a prognostic marker has been demonstrated previously [33-35]. In vivo fundus autofluorescence imaging allows visualization of metabolic changes on the level of the RPE cell monolayer [36-39] and therefore provides information beyond conventional fundus photography or fluorescein angiography. In essence, dominant fluorophores in lipofuscin granules of the RPE cell monolayer are recorded, whereby lipofuscin accumulates with age and in association with various complex and monogenetic retinal diseases. Recently, it has been shown that by means of fundus autofluorescence imaging different phenotypic patterns of abnormal fundus autofluorescence in the junctional zone of late atrophic AMD can be identified [34]. Moreover, there was a high degree of intra-individual symmetry in the fundus autofluorescence pattern in the two eyes of individual patients, but a high degree of inter-individual variability which may suggest genetic heterogeneity. In a preliminary analysis, seven AMD patients exhibiting the fundus autofluorescence pattern "diffuse-fine granular with peripheral punctate spots" (resembling Stargardt's macular dystrophy; age of onset, 50-84 years) and 14 GA patients exhibiting other fundus autofluorescence patterns were screened for *ABCA4* mutations. In the first group, all patients showed at least one mutated allele, and in two patients, two mutated alleles were detected. In the control group of 14 AMD patients exhibiting GA, but a different pattern of abnormal fundus autofluorescence, only two patients showed one mutated allele [40]. We suggest that this distinct AMD phenotype exhibiting "diffuse-fine granular with peripheral punctate spots" reflects genetic alterations in *ABCA4* and we speculate this distinct phenotype represents late onset Stargardt's macular dystrophy mimicking atrophic AMD. These preliminary data suggest that refined phenotyping is paramount in dissecting the role of candidate genes.

### Linkage and association studies in age-related macular degeneration

Over the past several years, researchers have carried out both linkage studies and association studies in an attempt to identify the genomic regions containing susceptibility loci for AMD. While linkage studies search for genetic markers that segregate with the disease in a familial constellation, association analyses identify genetic marker alleles that either cause disease or are in strong linkage disequilibrium (LD) with the disease-causing alleles.

Fisher and colleagues applied the genome-scan meta-analysis (GSMA) method that allows linkage results from several studies to be combined, providing greater power to identify regions which show only weak evidence for linkage in individual studies [41]. This method has been successful in a number of complex diseases and was applied to six published AMD genome-wide linkage scans: (1) Abecasis et al. [42], (2) Iyengar et al. [43], (3) Majewski et al. [44], (4) Schick et al. [45], (5) Seddon et al. [46], and Weeks et al. [47]. For each study, 120 genomic bins of 30 cM were defined and ranked according to maximum evidence for linkage within each bin. Bin ranks were weighted according to study size and summed across all studies. A high summed rank indicates a region with consistent evidence for linkage across studies. The strongest evidence for an AMD susceptibility locus was found on chromosome 10q26 where genome-wide significant linkage was observed (p=0.00025). Several other regions met the empirical significance criteria for bins likely to contain linked loci including adjacent pairs of bins on chromosomes 1q, 2p, 3p, and 16. Several of the regions identified here showed only weak evidence for linkage in the individual studies. The analysis performed by Fisher and colleagues may help prioritize regions for future positional and functional candidate gene studies in AMD.

### Complement factor H gene

Genome-wide linkage analyses and the genome-scan meta-analysis of Fisher and colleagues had pointed to a locus on 1q25-q31 [42-44,46-48]. Case-control studies recently identified complement factor H (*CFH*) as the responsible gene [49-52]. The *CFH* Y402H variant, located within a binding site for C-reactive protein (CRP), has consistently been shown to reveal strong association with AMD [53-55].

In a population-based prospective design on a total of 5681 individuals, investigators of the Rotterdam Eye Study have shown that *CFH* is implicated in all stages of AMD from early hallmarks such as drusen to vision-disabling late AMD [56]. The risk increases with each successive stage to an odds ratio of 11.0 for late AMD. It was calculated that individuals homozygous for the *CFH* Y402H polymorphism have a 48% risk of developing late AMD by age 95 years while this risk does not exceed 22% for non-carriers. Interestingly, complement factor H was associated with both late AMD subtypes (CNV and GA) in this study. Homozygous *CFH* Y402H carriers had a higher risk of bilateral than of unilateral late AMD, and risks of GA and mixed AMD were slightly but not significantly higher than neovascular AMD. This is in agreement with other studies that reported higher frequencies of CFH Y402H carriers in persons with GA [54,57] and one study that suggested a lower risk of GA for a *CFH* haplotype containing the non-risk allele [52]. In a comprehensive survey including variants from three gene loci (*CFH*, *LOC387715/HTRA1*, and *C2-FB*), Maller and co-workers did not find any association with phenotypic subclassifications of late AMD despite good power to detect association [58]. These findings suggest that the high risk for both subtypes of late AMD signifies a common pathogenesis involving the complement system.

CFH is an important regulator of the complement system. Three enzyme cascades exist (see e.g. figure 5 in [59]): the classical complement pathway, initiated by antigen-antibody complexes and surface-bound CRP; the lectin pathway, turned on by mannose groups of microbial carbohydrates; and the alternative complement pathway, activated by surface-bound C3b. The pathways converge at the point where C3 is cleaved into C3a and C3b by C3 convertase, which initiates C5 convertase, finally resulting in the formation of the membrane attack complex with the terminal components (C5b-C9). CFH specifically inhibits the alternative complement cascade but also regulates the common pathway. It binds C3b and acts as a cofactor in the proteolysis of C3b by factor I, resulting in an inactive C3b molecule. This prevents the production of C3 convertase in the alternative cascade as well as the production of C5 convertase in the common pathway. As a result, *CFH* interferes with the progression of the entire cascade [60-63]. Indeed, Hageman and co-workers showed that CFH and C3b/iC3b colocalize within drusen, suggesting that these regions represent complement activating surfaces within drusen and Bruch's membrane [52,63]. A recent study suggests that there may be multiple susceptibility alleles in the CFH genomic region with non-coding *CFH* variants possibly playing a role in disease susceptibility [64]. In this study, Li and co-workers examined the impact of 84 polymorphisms in a region of 123 kb overlapping the *CFH* gene on disease susceptibility in 544 unrelated affected individuals and 268 unrelated controls. As expected, strong association was observed between disease status and the Y402H-encoding variant (rs1061170). Unexpectedly, 20 other variants showed even stronger association. The strongly associated SNPs fell into two LD groups. The Y402H-encoding variant was included in one of the LD groups. The three SNPs showing strongest association were a synonymous SNP in exon 10, rs2274700, and two intronic SNPs, rs1410996, and rs7535263. The authors conclude that multiple haplotypes in the genomic region seem to modulate the AMD disease risk and that there are multiple disease-predisposing variants. Because the polymorphisms showing the strongest association with AMD susceptibility appear not to effect a change in the CFH protein, the authors speculate that these variants may be important in regulating the expression of *CFH*, or other nearby complement genes or both [64].

The region which includes the *CFH* gene cluster also contains numerous CFH-like genes (e.g. *CFHR1, CFHR2, CFHR3, CFHR4, and CFHR5*), which reveal high sequence conservation making any analysis difficult. Hughes and colleagues genotyped polymorphisms spanning the *CFH* gene cluster in 173 individuals with severe neovascular AMD and 170 controls and found a common haplotype, GTATAAAG, associated with decreased risk of AMD which was present on 8% of chromosomes of AMD patients and 20% of chromosomes of controls [65]. They found that this haplotype carried a deletion of *CFHR1* and *CFHR3*. Protein blot analysis of serum samples from individuals homozygous for each haplotype confirmed the absence of CFHR1 and CFHR3 protein. *CFHR1* and *CFHR3* proteins usually are present in the circulation and have the potential to compete with CFH for C3 binding. Possibly, CFH produced from full-length transcripts is beneficial regarding AMD-risk and other CFH-related proteins interfere with regulation of complement activity [65].

It has long been known that the prevalence of AMD varies widely among different ethnicities [66-70]. Moreover, the phenotypic spectrum of AMD among these groups is quite heterogeneous [71-75]. For example, in Japanese, soft drusen are only a moderate risk indicator (18%) for developing CNV compared with Caucasians, whereas serous pigment epithelial detachments are a very common high risk indicator (58%) for developing CNV in Japanese [75]. To explore the ethnic variation of the frequency of the *CFH* Y402H sequence variant, Grassi and co-workers analyzed the frequency of the risk (C) allele in populations from five different ethnicities. Widely divergent frequencies were noted between some of these populations (7-35%; Table 3).

**Table 3:** Prevalence of the histidine alteration in five different population from different ethnicity.

	**Caucasian**	**African-American**	**Hispanic**	**Japanese**	**Somali**
C	0.34 (0.03)	0.35 (0.04)	0.17 (0.03)	0.07 (0.02)	0.34 (0.03)
T	0.66 (0.03)	0.65 (0.04)	0.83 (0.03)	0.93 (0.02)	0.66 (0.03)
CC	0.07 (0.02)	0.11 (0.03)	0.05 (0.02)	0.02 (0.02)	0.07 (0.02)
CT	0.54 (0.04)	0.48 (0.06)	0.25 (0.05)	0.09 (0.03)	0.55 (0.04)
TT	0.39 (0.04)	0.41 (0.06)	0.70 (0.05)	0.89 (0.03)	0.38 (0.04)
Total patients	148	75	81	82	128

Values are frequency (standard error) [94].

These data suggest that there are other as yet unidentified genetic factors important in the pathogenesis of AMD. These factors may operate independently or mitigate the affects of the *CFH* Y402H sequence variant. Specifically, the findings suggest the presence of additional genetic risk factors for AMD in Japanese individuals.

### LOC387715/HTRA1

**Figure 2:**
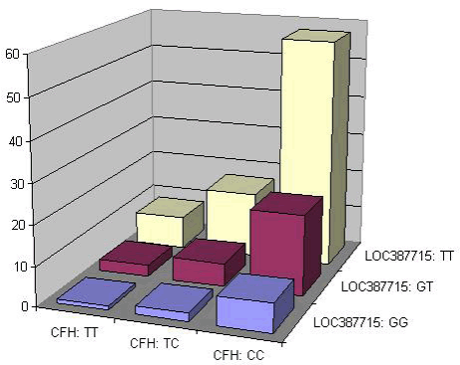
Two-locus (*LOC387715* and *CFH*) genotype specific disease risks. Two-locus genotype specific disease risks for the two variants: *LOC387715* (A69S) and *CFH* (Y402H) according to Rivera et al. [57].

Two recent reports have highlighted the *LOC387715/HTRA1* locus within 10q26 as a second major locus contributing to AMD pathogenesis [57,76]. Rivera and co-workers found the strongest association over the *LOC387715* gene conferring a 7.6-fold increased risk for individuals homozygous for a potential non-synonymous coding SNP, Ala69Ser. These findings were fully replicated in an independent case-control cohort. Furthermore, they replicated the strong association of AMD with the Y402H coding variant in *CFH*. The results indicate an independent contribution of the effects of risk alleles at the *LOC387715* (Ala69Ser) and *CFH* (Tyr402His)-gene locus to the overall disease risk (Figure 2). Very recently, the findings have been independently replicated by others [58,77-79].

**Figure 3:**
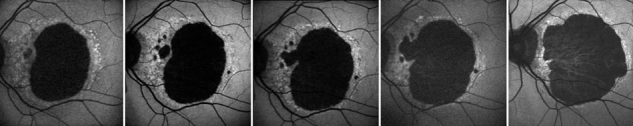
Progression of geographic atrophy imaged by fundus autofluorescence. Fundus autofluorescence images obtained in 12-month intervals in an AMD patient with a cSLO (Heidelberg retina angiograph, HRA classic and HRA 2, Heidelberg Engineering, Dossenheim, Germany). A large kidney-shaped area of GA was present at baseline (left) corresponding to decreased fundus autofluorescence (dark area). Recovered in yearly intervals, the area of the central atrophic area increased [80].

Patient groups of early high-risk AMD and late AMD were not different in risk allele distribution in *LOC387715*. This was also true for GA and neovascular AMD. So far, it is unknown, whether risk alleles at *LOC387715/HTRA1* as well as *CFH* correlate with severity stage of AMD or with a clinical outcome measure that would be a target of therapeutic intervention. Based on longitudinal data of serial fundus autofluorescence images from patients with late atrophic AMD, it has become feasible to determine the progression of GA in individual patients (Figure 3) [35,80].

The GA progression rate represents both a biologically based quantitative phenotype of late AMD and the most relevant target for therapeutic intervention. In a preliminary analysis, we determined whether the risk alleles of both *CFH* and *LOC387715/HTRA1* are correlated with the progression of GA in 207 AMD patients with GA (without any signs of CNV). We found that the risk allele distribution of Y402H in *CFH* and A69S in *LOC387715/HTRA1* for patients with GA is similar to those previously reported for pooled AMD samples. However, no correlation was found between the rate of progression of GA and *CFH* and/or *LOC387715/HTRA1* genotype [81]. These data suggest that both genes contribute to the increased risk of late AMD largely or entirely through their impact on precursors (such as drusen and/or other RPE/Bruch's membrane changes). This may have implications for therapeutic interventions in patients with late AMD, because the attempt to modify the respective gene products may not be promising [81].

DeWan and colleagues performed a genome-wide association study in 96 Chinese patients with neovascular AMD and 130 controls and confirmed a significant association with the A69S (rs10490924) polymorphism at the *LOC387715/HTRA1* locus within 10q26 [82]. A more telomeric SNP, rs11200638, was identified to be in almost complete linkage disequilibrium with rs10490924. SNP rs11200638 is localized in the putative GC-rich promoter region of the *HTRA1* gene potentially modulating expression levels of the gene [82]. Yang and co-workers provided similar findings in 581 Caucasian AMD patients and 309 controls [83]. DeWan et al. conclude that HTRA1 influences specifically CNV formation in AMD pathogenesis [82], although this conclusion seems unsubstantiated as exclusively wet AMD was included in their study. Unfortunately, the study of Yang et al. does not provide any information about the study phenotypes [83]. Obviously, such studies would considerably benefit from more extensive phenotypic analyses.

### Factor B/complement component 2

Recent findings have demonstrated the validity of the candidate gene approach given the pre-existing knowledge that the complement system plays a significant role in AMD pathogenesis. Gold and colleagues reported an association with two other genes that encode regulatory proteins acting along the same biological pathway as *CFH* [84]. These two genes are factor B (*BF*) and complement component 2 (*C2*), located 500 base pairs apart on chromosome 6p within the major histocompatibility complex class III region. The reported association was found in a sample of 898 patients with various forms of AMD and 389 controls. There was a common risk haplotype across *BF* and *C2* (OR, 1.32), as well as two protective haplotypes (OR, 0.36, and 0.45, respectively) [84]. These data have been independently replicated [58].

Backing the statistical data, *BF* and *C2* expression was shown in the neural retina, RPE and choroid. BF protein was present in ocular drusen and Bruch's membrane and less prominently in the choroidal stroma. The distribution of BF was similar to that of C3, both of which are similar to that of CFH and C5b-9 [84].

### Gene-gene and gene-environment interaction in AMD

In a comprehensive survey of variants at *CFH*, *LOC387715/HTRA1* and *C2-FB* in 2.172 unrelated individuals (1.238 affected individuals and 934 controls), Maller and co-workers developed a risk model for AMD based on five validated common variants. In contrast to the modest elevation in overall risk to siblings (two- to sixfold [9,10,13]), the predictive value of specific genotype combinations was notable. For example, approximately 10% of the population have a 40-fold greater risk and 1% (high-risk homozygotes at all three loci) have a more than 250-fold increased risk compared to baseline which is observed for individuals carrying the lowest-risk genotypes at all three loci (approximately 2% of the population) [58]. When evaluating the role of gene-gene interaction (epistasis) among the five common variants at the three loci (*CFH*, *LOC387715/HTRA1*, and *C2-FB*), statistically significant non-additive interactions were not found despite excellent power to detect epistasis. Specifically, a model in which the risk alleles at the three loci act independently (individual risks are multiplied to generate a combined risk profile) provided a better fit of the observed data than the same model with the inclusion of interlocus interference [58]. Similarly, the study of Rivera et al. indicated that the two risk alleles, *CFH* Y402H and *LOC387715* A69S, independently contribute to disease risk. Fitting an interaction model between *CFH* and *LOC387715*, no evidence of epistasis was found [57]. Conley et al. also found an independent multiplicative effect of *CFH* and *LOC387715* without significant interaction in two independent cohorts [78].

Several environmental factors have been identified over the past decade including cigarette smoking [85-88], higher body mass index (BMI) [89,90], and nutritional factors [91,92], with smoking being the most consistent in several population based studies worldwide [85,86]. So far, however, there are inconclusive data on gene-environment interactions. In an extended collection of 848 AMD cases, no significant differences in risk allele frequency for either *CFH* or *LOC387715* were detected between smokers and non-smokers despite substantial power by Rivera et al. [57], whereas Schmidt and co-workers observed significant evidence for a statistical interaction between the *LOC387715* A69S variant and a history of cigarette smoking [77]. Despriet and colleagues found that the combined effect of homozygosity for the Y402H variant in *CFH* and smoking exceeds the sum of the independent effects. Compared with no exposure, smoking increased the risk of AMD 3.3 times, the presence of two *CFH* Y402H alleles increased the risk 12.5 times, while the combination of both determinants increased this risk 34-fold [56]. In contrast, Conley and co-workers excluded a significant interaction of risk allele distribution in CFH or LOC3897715 and cigarette smoking [78]. Similarly, Seddon and co-workers did not find a statistically significant interaction between *CFH* genotype and cigarette smoking, but the susceptibility to late AMD was modified by the body mass index (BMI; normal values according to WHO, 2000 EK IV: 18,5 kg/m^2^; -25,0 kg/m^2^;). Compared with lean individuals with the *CFH* TT genotype, an increased risk of AMD among these lean individuals with BMI lower than 25 was found only for the CC homozygotes. For heavier persons with BMI greater than 25, the risk varied from a non-significant null or slightly protective association for the TT genotype, to a moderately high 2.2-fold increased risk for the heterozygotes, and a very high 5.9-fold increased risk for the CC homozygote state. This interaction between BMI and genotype related risk of late AMD was statistically significant for the CT versus TT genotype [93].

## Conclusions

Genetic studies have convincingly demonstrated that there exist common alleles of substantial effect on AMD pathogenesis. The finding of such common alleles with substantial effects makes predictive DNA testing a tempting option although the mechanisms and thus the biological consequences conferred by the common risk alleles at the respective gene loci are not yet understood. Consequently, the knowledge of being carrier of risk alleles is currently not matched by adequate options for preventive strategies or possible treatment modalities.

The finding that variants within *CFH* and *BF* are responsible for a large fraction of AMD cases (at least in Caucasians) suggests an important role of the alternative complement pathway in the pathobiology of AMD and further strengthens the notion that inflammation has a major role in this common disease [84].

So far, the identification of genetic factors has not resulted in therapeutic strategies to modify the disease. The data on gene-environment interactions are inconclusive and gene-gene interactions have not been observed despite substantial power. Other genetic factors most likely have yet to be discovered. These, in combination with environmental variables, will further stratify individual risk more accurately. A correlation between the common genetic risk variants and clinical outcome measures (e.g. the progression of GA) has not been observed. The role of rare variants is still obscure. Especially for the latter, refined phenotyping may be paramount.

Strategies to elucidate the genetic influence on AMD may include (1) the search for additional susceptibility genes, (2) the examination of the role of the known common variants (especially at *CFH* and *LOC387715/HTRA1*), (3) the investigation of gene-environment and gene-gene interaction and (4) the identification of modifying genes. To achieve these goals, large cohorts of phenotypically well characterized subcategories will be needed. Biologically based quantitative phenotyping is required to increase the power of linkage and association studies. Because AMD is a complex disease, individual gene effects might only be detected within subgroups of patients with specific environmental exposures. Environmental factors for stratification include cigarette smoking and body mass index. Also, a given gene variant might only result in a detectable phenotype when acting in combination with additional susceptibility alleles either additively or multiplicatively. Additional work exploring these types of interactions should bring us closer to the genes influencing the onset and progression of AMD.
